# Empathy and Theory of Mind in Multiple Sclerosis: A Meta-Analysis

**DOI:** 10.3389/fpsyt.2021.628110

**Published:** 2021-04-09

**Authors:** XiaoGuang Lin, XueLing Zhang, QinQin Liu, PanWen Zhao, JianGuo Zhong, PingLei Pan, GenDi Wang, ZhongQuan Yi

**Affiliations:** ^1^Department of Neurology, Affiliated Suqian Hospital of Xuzhou Medical University, Suqian, China; ^2^Department of Central Laboratory, Affiliated Yancheng School of Clinical Medicine of Nanjing Medical University, Yancheng, China; ^3^Department of Neurology, Affiliated Yancheng School of Clinical Medicine of Nanjing Medical University, Yancheng, China; ^4^Department of Neurology and Department of Central Laboratory, Affiliated Yancheng School of Clinical Medicine of Nanjing Medical University, Yancheng, China

**Keywords:** multiple sclerosis, empathy, theory of mind, meta-analysis, cognitive, affective

## Abstract

Multiple sclerosis (MS) is an immune-mediated demyelinating disease of the central nervous system. Studies have shown that MS disrupts several social cognitive abilities [including empathy and theory of mind (ToM)]. Overall ToM deficits in MS are well documented, but how the specific ToM subcomponents and empathic capacity are affected remains unclear. For this meta-analysis, we searched PubMed, Web of Science, and Embase from inception to July 2020. Effect sizes were calculated using Hedges *g* with a random-effects model. Thirty-three studies were included. Relative to healthy controls (HCs), patients with MS were moderately impaired in overall empathy (*g* = −0.67), overall ToM (*g* = −74), cognitive ToM (*g* = −0.72), and the overlapping domains of cognitive empathy/affective ToM (*g* = −0.79); no group differences were identified for affective empathy (*g* = −0.19). Compared with HCs, patients with relapsing-remitting MS (RRMS) and progressive MS were impaired in overall empathy, overall ToM, cognitive ToM, and cognitive empathy/affective ToM, without significant RRMS–progressive MS differences in impairment degree. We conducted the first meta-analytic review investigating the empathy and ToM functioning patterns in patients with MS and examined the overlapping and distinct subcomponents of these constructs. The findings suggest differential impairment of the core aspects of social cognitive processing in patients with MS, which may importantly inform the development of structured social cognitive MS interventions.

## Introduction

Multiple sclerosis (MS) is an immune-mediated demyelinating disease of the central nervous system (1), which is characterized by multifocal destruction of the myelin sheath and axonal loss (2, 3). Patients usually develop sensorimotor, visual, and emotional symptoms as well as cognitive impairment, leading to functional disability and reduced quality of life (QoL) (4). The precise etiology of MS remains unclear, and the prognosis is variable and unpredictable.

Cognitive impairment has been recognized as a common symptom in MS, with an estimated lifetime occurrence of 40–65% (5–7). The cognitive domains generally affected include executive functioning, information processing speed, attention, and memory (8, 9), and social cognition (10–14). Social cognition, a basic means for the individual to perceive, encode, store, retrieve, and regulate information regarding other people and the self (15), has a remarkable impact on interpersonal communication and QoL (16–19). Social cognition is a multidimensional construct, mainly involving four dimensions: empathy, theory of mind (ToM), social perception and social knowledge, and attribution bias (15, 17).

One core aspect of social cognition, i.e., empathy, refers to the ability to understand and identify the mental states of others, as well as the ability to share the feelings of others (20). It is a complex construct with multiple components, usually including affective and cognitive domains. Emotional empathy is described as “I feel your feelings” and can be regarded as primitive empathy, while cognitive empathy refers to “I understand your feelings” and can be regarded as advanced empathy (21–23). This is significant in clinical practice, as any deficit in cognitive or affective empathy can lead to atypical emotional reactions, but the clinical treatment implications differ (20, 24). Recently, several studies have assessed empathy deficits in patients with MS with inconsistent findings. For example, Realmuto et al. (25) and van der Hiele et al. (26) found no differences between patients with MS and healthy controls (HCs) in terms of empathy, whereas Kraemer et al. (27) found moderate impairment in empathy in patients with MS compared to HCs. These inconsistent findings may be related to low statistical power, as many of these studies enrolled small sample sizes. To answer important clinical questions, a quantitative meta-analysis is needed to test the magnitude and significance of empathy in MS to increase the statistical power and refine the conclusions derived from the inconsistent findings of the previous studies.

ToM, another core domain of social cognition, refers to the ability to attribute mental states (beliefs, intentions, and desires) to others and to use the attributions to understand and predict behavior (28, 29). Like empathy, ToM can also be divided into affective and cognitive components (30). Affective ToM refers to the capacity to understand others' emotional states, and cognitive ToM is the ability to infer other's thoughts, intentions, and beliefs (31). To our knowledge, two recent meta-analyses examined ToM differences between patients with MS and HCs. Cotter et al. and Bora et al. calculated the overall ToM score based on numerous different ToM tasks (a combination of affective ToM and cognitive ToM tasks) and found that patients with MS have ToM deficits (32, 33). However, it remains unclear whether these defects were attributable to only one or both subcomponents, as no specific subgroup analysis was conducted for affective and cognitive ToM.

Notably, although there are differences between cognitive empathy and affective ToM in definition (34), these two constructs are difficult to distinguish at a purely behavioral level of assessment because they both involve attribution of another's emotional states (35). Additionally, overlap between cognitive empathy and affective ToM has often been noted (24, 36, 37). Therefore, in this study, we considered cognitive empathy and affective ToM to be interchangeable.

To this end, the present study aimed to provide the first meta-analytic integration of broader empathy and ToM in MS with the affective and cognitive subcomponents of both these abilities distinguished. Moreover, specific subgroup analyses for the overlapping components (cognitive empathy and affective ToM) and separate components (cognitive ToM and affective empathy) were also conducted. Besides, considering that MS is a heterogeneous disease, with subtypes and diverse trajectories, in influx between relapse, remission, stability, and progression (38, 39), we performed subgroup analyses (including of relapsing-remitting MS [RRMS] and progressive MS (including progressive primary MS and secondary progressive MS).

In addition, studies have reported that certain clinical behavioral symptoms may have a significant relationship with social cognition (40–44). Clinically, depression and anxiety are common behavioral symptoms in patients with MS (45–50), and it has been reported that social cognitive deficits are significantly related to the severity of depressive symptoms or anxiety symptoms in some diseases (40–42, 44). So far, the exact relationship between social cognitive performance and the severity of depression or anxiety in patients with MS remains unclear. Therefore, we evaluated the effect of potential variables [such as sex (ratio of female patients in the MS group), mean age, education level, disease duration, Expanded Disability Status Scale (EDSS) score, quality assessment score, severity of depression, and severity of anxiety] on social cognition. With this meta-analysis, we hope to promote a more comprehensive and nuanced understanding of how these two core domains of social cognition are affected in MS.

## Methods

### Study Registration

This study was performed per the Preferred Reporting Items of Systematic Review and Meta-Analysis (PRISMA) guidelines (51). This protocol was prospectively registered at the International Platform of Registered Systematic Review and Meta-analysis Protocols (ID: INPLASY202070029) and has been released in the journal of Medicine (52).

### Data Sources and Study Selection

A systematic literature search was conducted across the PubMed, Web of Science, and Embase databases from inception to July 2020. The following search terms were used: “multiple sclerosis” or “MS” or “clinically isolated syndrome” combined with: “social cognition” or “theory of mind” or “ToM” or “mentalizing” or “mentalizing” or “Reading the Mind in the Eyes Test” or “Faux pas task” or “False Belief” or “the Awareness of Social Inference Test” or “Virtual Assessment of Mentalising Ability” or “the Movie for the Assessment of Social Cognition” or “picture sequencing task” or “Cartoon Test” or “Hinting Test” or “Strange Stories Test” or “facial expression^*^” or “prosody” or “pragmatic impairment” or “non-literal language” or “sarcas^*^” or “lie^*^” or “joke^*^” or “empath^*^” or “perspective taking” or “Peer-Report Social Functioning Scale.” Furthermore, other resources, such as the reference lists of all included studies, were searched manually.

### Inclusion Criteria

Studies were included if they met four criteria. First, the study should have compared patients with MS to a matched HC group. Second, the study should have assessed empathy performance or ToM performance using standard measures. Third, the study should have provided sufficient data to calculate the effect sizes of empathy or ToM. Fourth, the study should have been published in a peer-reviewed journal in English.

### Exclusion Criteria

Studies were excluded for three reasons. First, if the participant overlapped with a participant in another study with a larger sample size. Second, if they lacked an HC group. Third, if they included <10 participants to ensure the reliability of the outcome (29).

### Screening and Data Extraction

Article retrieval, screening, data extraction, and quality evaluation were independently completed by two investigators. The relevant data extracted included: (a) Title information, such as first author, publication year, and title; (b) Sample characteristics from the MS and HC groups, such as sample size, sex (female and male), mean age, education level, disease duration, EDSS scores, severity of depression, and severity of anxiety; (c) For both empathy and ToM, tasks were divided into affective and cognitive subcomponents, and the classification was based on the nature of the task and the information provided by the author of the original article; (d) The data used for calculating the effect sizes of empathy or ToM. Any disagreements were first discussed between these two investigators, and further disagreements were arbitrated by a third investigator.

### Study Quality Assessment

To assess study quality, a nine-star protocol was used based on the Newcastle-Ottawa Scale for case-control studies. Studies with ≥7 stars were considered high-quality (53).

### Statistical Analysis

Meta-analyses were conducted using the Stata 15.0 software package (54). The effect size (Hedges *g*) and 95% confidence interval (CI) were calculated to estimate differences in ToM and empathy between the MS and HC groups (55). The magnitude of Hedges *g* could be interpreted using Cohen *d* effect size conventions, and effect sizes were deemed small, moderate, or large when their values were equal to or larger than 0.2, 0.5, or 0.8, respectively (56).

When studies did not provide a total mean score on a particular measure but reported subscores (i.e., individual ToM tasks presented separately), pooled effect sizes were aggregated by computing the mean effect size (and standard error) (57). Similarly, when studies reported the effect size per subgroup [i.e., by clinical subtypes (relapsing MS and progressive MS)], data were pooled into an overall effect size (57). Meta-analyses were completed using a random-effects model, as it better accommodates heterogeneous effect distributions.

The degree of heterogeneity within effect size estimates was tested with the *I*^2^ statistic, and the degree of heterogeneity was deemed low, moderate, or large when *I*^2^ was equal to or larger than 0, 50, or 75%, respectively (58).

To assess the risk of publication bias, Egger's test was used. For this analysis, significance indicates that bias may be present [*p* < 0.05; (59)]. Additionally, the trim-and-fill analyses were applied, providing effect sizes adjusted for publication bias (60).

Meta-regression analyses were conducted to investigate whether demographic and clinical variables (including age, sex, education level, disease duration, and EDSS score, quality assessment score, severity of depression, and severity of anxiety) explained the variance in any of the effects identified. As a measure of severity of depression or anxiety, according to accepted cut-off scores of depression or anxiety rating scales used, studies were classified as no symptoms = 0, mild symptoms = 1, moderate symptoms = 2, severe symptoms = 3 (41, 61–63). For each of these analyses, a minimum of 10 data points was required for each relevant predictor variable and the social cognitive ability under assessment (64).

## Results

### Study Characteristics

**Figure 1:**
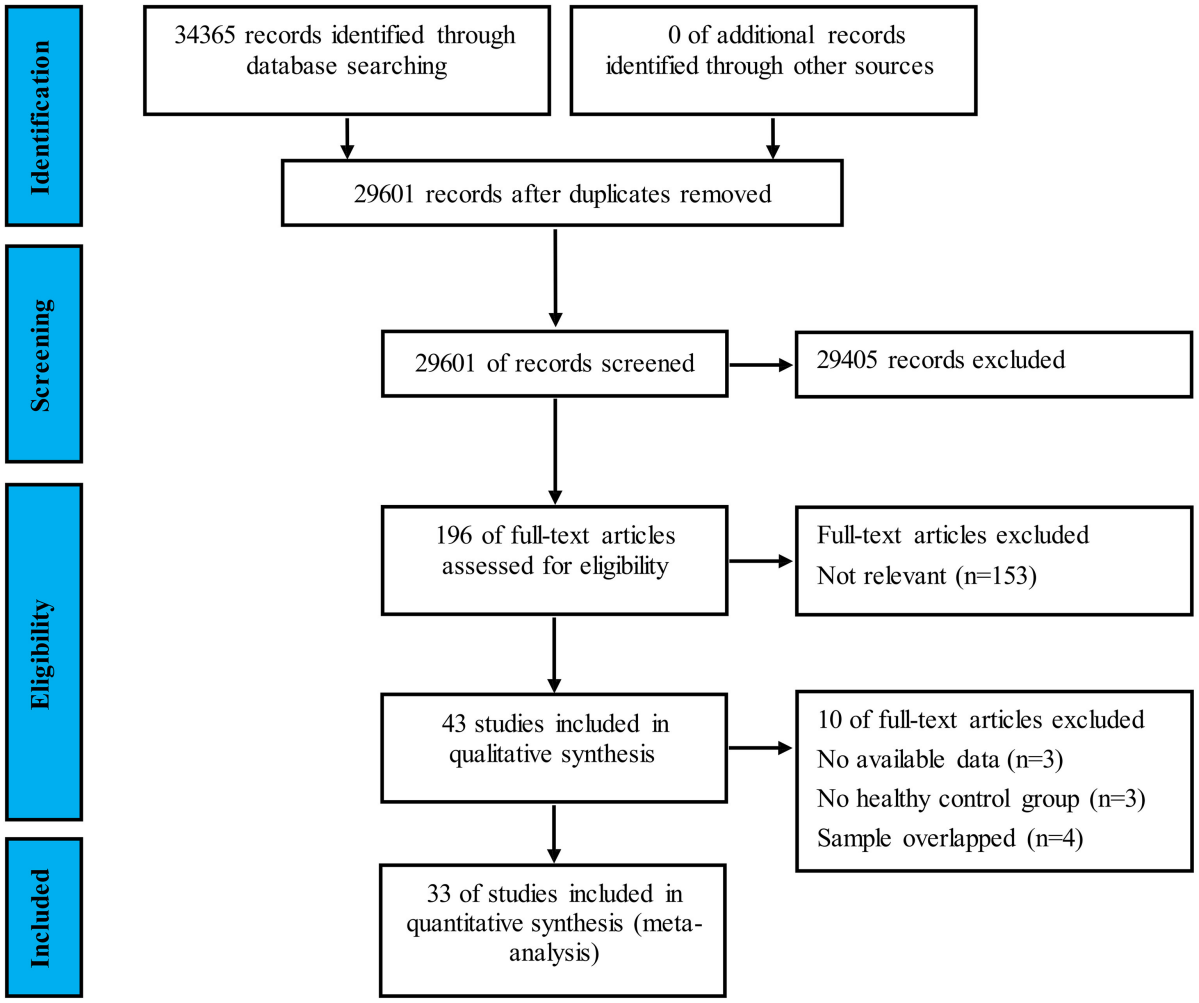
PRISMA flowchart displaying study screening and selection process.

The flow chart of the study selection process is shown in Figure 1. In total, 34,365 potentially eligible articles were retrieved. After the removal of duplicates, 29,601 articles remained, which were then subjected to title and abstract screening. Of these, 43 initially met the inclusion criteria. Three of these studies did not include an HC group (65–67); another three were excluded for lack of sufficient data to calculate the effect sizes and standard errors of empathy or ToM (18, 68, 69). Four studies were excluded, as their samples overlapped with those of other studies (14, 70–72). Eventually, 33 studies with 1,568 patients with MS (mean age = 40.71 years, *SD* = 9.63 years, 70.4% female) and 1,283 HCs (mean age = 39.18 years, *SD* = 9.91 years, 65.3% female) were included in the meta-analysis [Table 1; (4, 11–13, 25–27, 46, 48, 50, 63, 73–94)].

**Table 1:** Individual study characteristics.

**Study**	**HCs groups**		**MS groups**				**Task**	**Type**
	**Sample (female)**	**Age (years, SD)**	**Sample (female)**	**Age (years, SD)**	**Duration (years, SD)**	**EDSS (SD)**		
Banati et al. (63)	35 (18)	33.40 (7.80)	40 (29)	36.20 (9.40)	NA	NA	Faux pas task	CogToM
							Adult Faces task	CogEmp/AffTom
							RMET	CogEmp/AffTom
							Baron-Cohen's EQ	Mixed Emp
Batista et al. (73)	60 (40)	36.10 (9.40)	60 (40)	37.20 (7.50)	10.6 (6.6)	2 (0.75)	RMET	CogEmp/AffTom
							ToM videos test	CogToM
Bisecco et al. (74)	25 (18)	37.83 (11.95)	41 (27)	34.18 (10.27)	8.8 (8.2)	2.375 (1.625)	RMET	CogEmp/AffTom
							ToM picture sequencing task	CogToM
Chanial et al. (75)	21 (16)	33.90 (7.00)	21 (15)	38.80 (5.50)	10 (7)	4.2 (2)	Faux pas task	CogToM
Charvet et al. (76)	32 (23)	15.69 (2.94)	28 (19)	16.29 (3.12)	2.82 (2.51)	1.5 (1)	RMET	CogEmp/AffTom
							Faux pas task	CogToM
							FB Task	CogToM
							Empathy Systemizing Quotient	Mixed Emp
Czekóová et al. (77)	43 (25)	34.70 (11.00)	43 (31)	35.80 (8.00)	7.5 (4.4)	2.5 (1.5)	RMET	CogEmp/AffTom
Dulau et al. (78)	60 (35)	43.20 (9.30)	60 (43)	46.50 (10.60)	14.4 (9.4)	3.5 (1.5)	Faux pas task	CogToM
							RMET	CogEmp/AffTom
							Attribution of intentions	CogToM
García et al. (79)	106	NA	35	NA	NA	NA	RMET	CogEmp/AffTom
Genova et al. (80)	15 (5)	38.90 (13.10)	15 (11)	49.50 (8.00)	17.98 (10.3)	NA	TASIT-SIE-Feel	CogEmp/AffTom
							TASIT-SIE-Do	CogToM
							TASIT-SIE-Say	CogToM
							TASIT-SIE-Think	CogToM
Genova and McDonald (13)	15 (11)	45.60 (11.70)	17 (9)	51.90 (9.20)	13.8 (9.5)	NA	TASIT-SIM	Mixed ToM
							TASIT-SIE	Mixed ToM
Gleichgerrcht et al. (81)	38 (33)	39.30 (8.10)	38 (33)	42.30 (11.30)	1.6 (8.7)	1.66 (1.6)	IRI Empathic Concern	AffEmp
							IRI Personal distress	AffEmp
							IRI Perspective Taking	CogEmp/AffTom
							IRI Fantasy	CogEmp/AffTom
Goitia et al. (82)	42 (29)	37.10 (10.70)	36 (30)	39.20 (10.20)	9.3 (7.3)	NA	Faux pas task	CogToM
Golde et al. (83)	30 (19)	39.57 (8.36)	30 (18)	40.20 (9.87)	8.23 (5.04)	1.875 (1)	MASC	Mixed ToM
							MET-Cognitive empathy	CogEmp/AffTom
							MET-Emotional empathy	AffEmp
Henry et al. (84)	30 (19)	44.30 (9.55)	27 (18)	47.00 (11.01)	7 (6.08)	1.9 (1.98)	RMET	CogEmp/AffTom
Henry et al. (4)	30 (21)	38.60 (13.90)	64 (50)	42.40 (9.80)	9.1 (5.37)	2.3 (1.7)	First-order FB task	CogToM
							Second-order FB task	CogToM
							Faux pas task	CogToM
Henry et al. (48)	33 (24)	43.70 (10.50)	62 (36)	46.80 (10.90)	11.4 (9.4)	3.8 (1.8)	First-order FB task	CogToM
							Second-order FB task	CogToM
							Faux pas task	CogToM
Ignatova et al. (50), EDSS < 3.5	36 (24)	42.40 (12.30)	18 (13)	41.90 (11.60)	7.06 (4.3)	1.86 (0.8)	RMET	CogEmp/AffTom
							Faux pas task	CogToM CogToM
							ToM cartoons	CogToM
							Self-Compassion Scale	Mixed Emp
Ignatova et al. (50), EDSS ≥ 3.5	36 (24)	42.40 (12.30)	18 (11)	43.70 (8.50)	11.17 (7.45)	4.56 (0.95)	RMET	CogEmp/AffTom
							Faux pas task	CogToM CogToM
							ToM cartoons	CogToM
							Self-Compassion Scale	Mixed Emp
Isernia et al. (85)	26 (19)	51.35 (12.42)	42 (24)	52.38 (10.31)	21.24 (10.94)	5.25 (1.75)	RMET	CogEmp/AffTom
							Faux pas task-Intention	CogToM
							Faux pas task-Emotion	CogEmp/AffTom
							Strange Stories-Double bluff	CogToM
							Strange Stories-White lie	CogToM
							Strange Stories-Misunderstanding	CogToM
							Strange Stories-Emotions	CogEmp/AffTom
							MASC-Thoughts	CogToM
							MASC-Intention	CogToM
							MASC-Affective	CogEmp/AffTom
Kraemer et al. (27)	25 (11)	33.44	25 (15)	30.92	1.24 (0.25)	0.94 (0.63)	MASC	Mixed ToM
							Baron-Cohen's EQ	Mixed Emp
Labbe et al. (86)	45 (22)	37.58 (12.00)	47 (26)	36.28 (10.21)	4.28 (3.65)	1.75 (1.25)	Faux pas task	CogToM
Lancaster et al. (87)	15	45.60 (11.67)	15	48.93 (8.60)	14.43 (9.09)	NA	VAMA cognitive	CogToM
							VAMA affective	CogEmp/AffTom
Mike et al. (88)	24 (13)	36.81 (7.27)	49 (31)	39.82 (9.31)	9.49 (6.19)	2.43 (1.71)	Faux pas task	CogToM
							Adult Faces task	CogEmp/AffTom
							RMET	CogEmp/AffTom
Neuhaus et al. (11)	34 (22)	43.90 (12.50)	35 (22)	43.80 (12.13)	12.9 (9.6)	3.125 (1.63)	Faux pas task	CogToM
							ToM cartoons	CogToM
							ToM-Inference test	CogToM
							RMET	CogEmp/AffTom
Ouellet et al. (46), MS–	20 (10)	48.50 (8.20)	26 (15)	45.20 (7.3)	10.2 (8.1)	3.8 (2.7)	Faux pas task	CogToM
							Strange Stories-Mental task	CogToM
							ToM-Conversations and Insinuations	Mixed ToM
Ouellet et al. (46), MS+	20 (10)	48.50 (8.20)	15 (12)	43.6 (8.3)	6.2 (4.6)	2.8 (2.2)	Faux pas task	CogToM
							Strange Stories	CogToM
							ToM-Conversations and Insinuations	Mixed ToM
Parada-Fernández et al. (89)	40 (20)	50.78 (10.08)	45 (29)	49.44 (9.44)	NA	NA	RMET	CogEmp/AffTom
Patil et al. (90)	38 (31)	39.30 (8.10)	38 (33)	42.3 (11.3)	10.6 (8.7)	1.66 (1.6)	IRI Empathic Concern	AffEmp
							IRI Personal distress	AffEmp
							IRI Perspective Taking	CogEmp/AffTom
							IRI Fantasy	CogEmp/AffTom
Pitteri et al. (12)	38 (28)	37.10 (8.90)	31 (24)	36.3 (7.6)	7 (4.5)	1 (0.875)	RMET	CogEmp/AffTom
							Baron-Cohen's EQ	Mixed Emp
Pöttgen et al. (91)	45 (31)	42.50 (10.45)	45 (31)	42.42 (10.66)	8.46 (6.18)	3.47 (1.63)	MASC-Thoughts	CogToM
							MASC-Intention	CogToM
							MASC-Affective	CogEmp/AffTom
Raimo et al. (92)	40 (31)	40.20 (11.40)	40 (29)	40.58 (11.51)	8.23 (7.48)	2.44 (1.48)	Advanced Test of ToM	CogToM
							ToM picture sequencing task	CogToM
							RMET	CogEmp/AffTom
							Emotion attribution task	CogEmp/AffTom
Realmuto et al. (25)	45 (32)	33.04 (7.73)	45 (31)	34.22 (7.35)	9.72 (6.22)	2.06 (1.46)	RMET	CogEmp/AffTom
							SET-identifying intentions	CogToM
							SET-emotional states	CogEmp/AffTom
Roca et al. (93)	16	40.88 (9.95)	NA	40.67 (9.53)	5.05 (3.75)	0.58 (0.99)	Faux pas task-Intention	CogToM
							Faux pas task-Emotion	CogEmp/AffTom
van et al. (26)	128 (94)	NA	278 (216)	NA			Baron-Cohen's EQ	Mixed Emp
Vanotti et al. (94)	53	36.40 (10.90)	121	42.3 (4.1)	3.53 (0.34)	2.1 (1.5)	RMET	CogEmp/AffTom

*SD, standard deviation; NA, not available; EDSS, Expanded Disability Status Scale; MS, multiple sclerosis; HCs, healthy controls; ToM, theory of mind; EMP, empathy; CogToM, Cognitive ToM; CogEmp/AffTom, Cognitive empathy/Affective ToM; AffEmp, affective empathy; FB, False Belief; RMET, Reading the Mind in the Eyes Test; IRI, Interpersonal Reactivity Index; TASIT, the Awareness of Social Inference Test; SIM, Social Inference-Minimal Test; SIE, Social Inference-Enriched; EQ, Empathy Quotient; MASC, the Movie for the Assessment of Social Cognition; MET, Multifaceted Empathy Test; VAMA, Virtual Assessment of Mentalizing Ability; SET, Story-based Empathy task*.

### Study Quality Assessment

The results of the study quality assessment are shown in Table 2. The mean score was 7.11 (*SD* = 0.83), and 29 of the 35 case-control studies were awarded ≥7 stars and considered of high quality.

**Table 2:** Quality evaluation of included studies.

**Study**	**S1**	**S2**	**S3**	**S4**	**C**	**E1**	**E2**	**E3**	**Sum**
Banati et al. (63)	⋆	—	—	⋆	⋆ —	⋆	⋆	⋆	6
Batista et al. (73)	⋆	—	⋆	⋆	⋆ ⋆	⋆	⋆	⋆	8
Bisecco et al. (74)	⋆	⋆	—	⋆	⋆ —	⋆	⋆	⋆	7
Chanial et al. (75)	⋆	⋆	—	⋆	— —	⋆	⋆	⋆	6
Charvet et al. (76)	⋆	⋆	⋆	⋆	⋆ —	⋆	⋆	⋆	8
Czekóová et al. (77)	⋆	⋆	—	⋆	⋆ —	⋆	⋆	⋆	7
Dulau et al. (78)	⋆	—	—	⋆	⋆ ⋆	⋆	⋆	⋆	7
García et al. (79)	⋆	—	—	⋆	— —	⋆	⋆	⋆	5
Genova et al. (80)	⋆	—	—	⋆	— ⋆	⋆	⋆	⋆	6
Genova and McDonald (13)	⋆	—	—	⋆	⋆ ⋆	⋆	⋆	⋆	7
Gleichgerrcht et al. (81)	⋆	⋆	—	⋆	⋆ ⋆	⋆	⋆	⋆	8
Goitia et al. (82)	⋆	—	⋆	⋆	⋆ ⋆	⋆	⋆	⋆	8
Golde et al. (83)	⋆	—	—	⋆	⋆ ⋆	⋆	⋆	⋆	7
Henry et al. (84)	⋆	—	⋆	⋆	⋆ ⋆	⋆	⋆	⋆	8
Henry et al. (4)	⋆	—	—	⋆	⋆ ⋆	⋆	⋆	⋆	7
Henry et al. (48)	⋆	—	—	⋆	⋆ ⋆	⋆	⋆	⋆	7
Ignatova et al. (50), EDSS < 3.5	⋆	—	—	⋆	⋆ ⋆	⋆	⋆	⋆	7
Ignatova et al. (50), EDSS ≥ 3.5	⋆	—	—	⋆	⋆ ⋆	⋆	⋆	⋆	7
Isernia et al. (85)	⋆	⋆	—	⋆	⋆ ⋆	⋆	⋆	⋆	8
Kraemer et al. (27)	⋆	—	—	⋆	⋆ ⋆	⋆	⋆	⋆	7
Labbe et al. (86)	⋆	—	—	⋆	⋆ —	⋆	⋆	⋆	6
Lancaster et al. (87)	⋆	—	—	⋆	⋆ ⋆	⋆	⋆	⋆	7
Mike et al. (88)	⋆	—	—	⋆	⋆ —	⋆	⋆	⋆	6
Neuhaus et al. (11)	⋆	—	—	⋆	⋆ ⋆	⋆	⋆	⋆	7
Ouellet et al. (46), MS–	⋆	—	—	⋆	⋆ ⋆	⋆	⋆	⋆	7
Ouellet et al. (46), MS+	⋆	—	—	⋆	⋆ ⋆	⋆	⋆	⋆	7
Parada-Fernández et al. (89)	⋆	—	—	⋆	⋆ ⋆	⋆	⋆	⋆	7
Patil et al. (90)	⋆	—	—	⋆	⋆ ⋆	⋆	⋆	⋆	7
Pitteri et al. (12)	⋆	—	—	⋆	⋆ ⋆	⋆	⋆	⋆	7
Pottgen et al. (91)	⋆	—	—	⋆	⋆ ⋆	⋆	⋆	⋆	7
Raimo et al. (92)	⋆	⋆	⋆	⋆	⋆ ⋆	⋆	⋆	⋆	9
Realmuto et al. (25)	⋆	—	—	⋆	⋆ ⋆	⋆	⋆	⋆	7
Roca et al. (93)	⋆	—	⋆	⋆	⋆ ⋆	⋆	⋆	⋆	8
van et al. (26)	⋆	⋆	⋆	⋆	⋆ ⋆	⋆	⋆	⋆	9
Vanotti et al. (94)	⋆	—	—	⋆	⋆ ⋆	⋆	⋆	⋆	7

*We herein selected “age” as the most important adjusting factor and selected “education level” as other controlled factor. S1, Is the case definition adequate?; S2, Representativeness of the cases; S3, Selection of Controls; S4, Definition of Controls; C, Comparability of cases and controls on the basis of the design or analysis; E1, Ascertainment of exposure; E2, Same method of ascertainment for cases and controls; E3, Non-Response rate. The ⋆ means one point. The — means no score*.

### Empathy and ToM in Patients With MS vs. HCs

**Figure 2:**
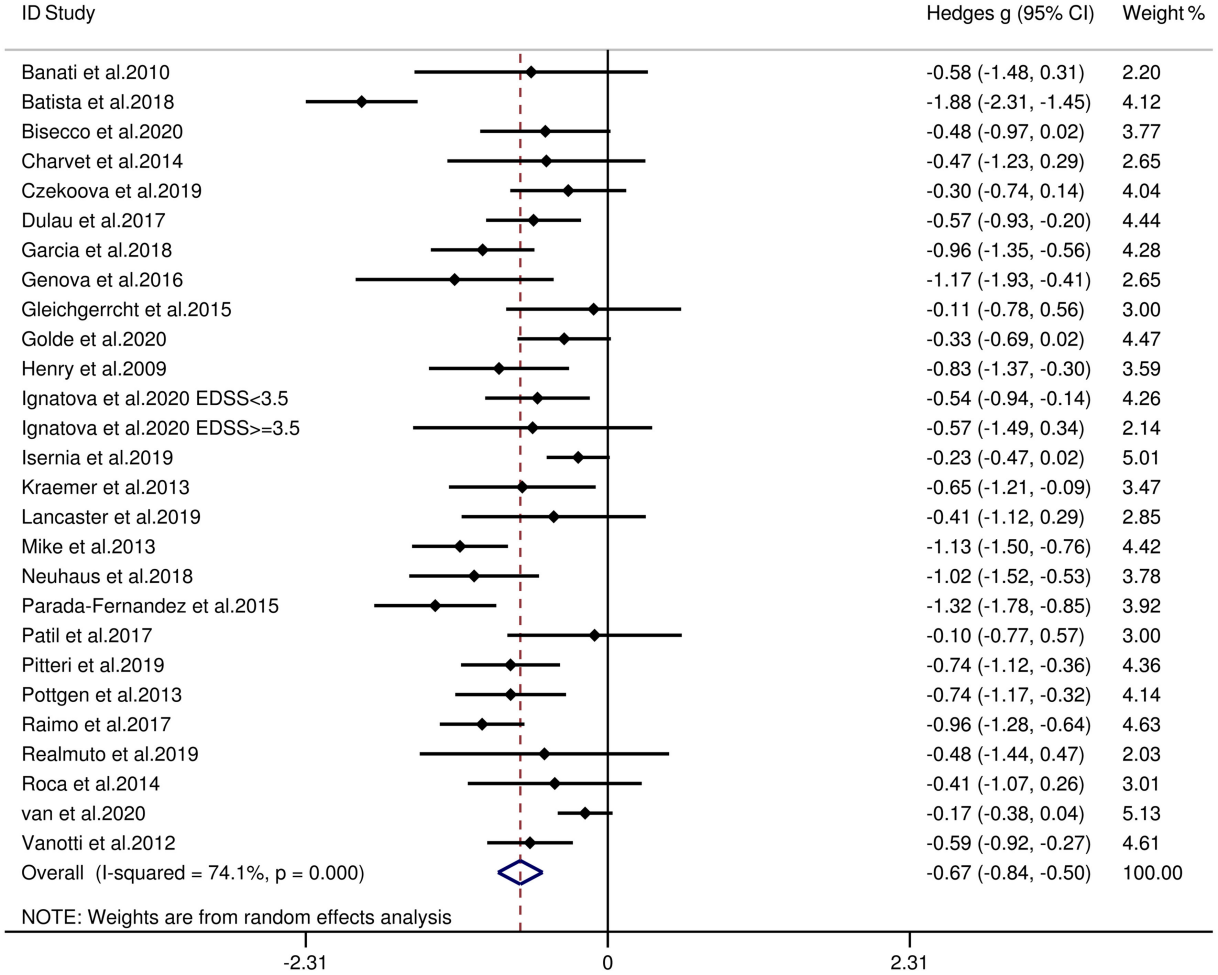
Forest plots showing effect size estimates (Hedges g) for overall empathy differences between MS and healthy controls. CI, confidence interval; MS, multiple sclerosis; ToM, theory of mind.

**Figure 3:**
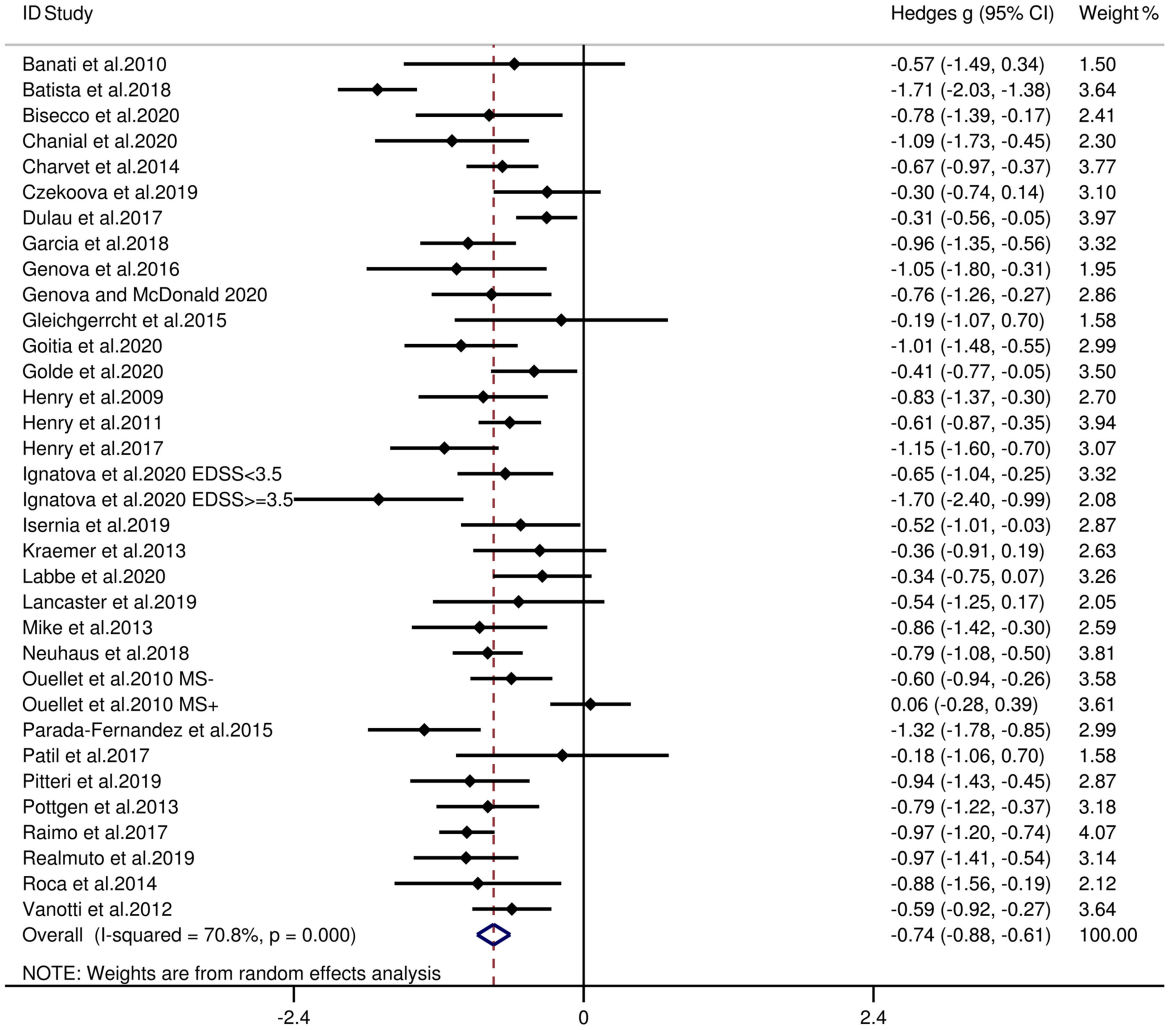
Forest plots showing effect size estimates (Hedges g) for overall ToM differences between MS and healthy controls. CI, confidence interval; MS, multiple sclerosis; ToM, theory of mind.

**Figure 4:**
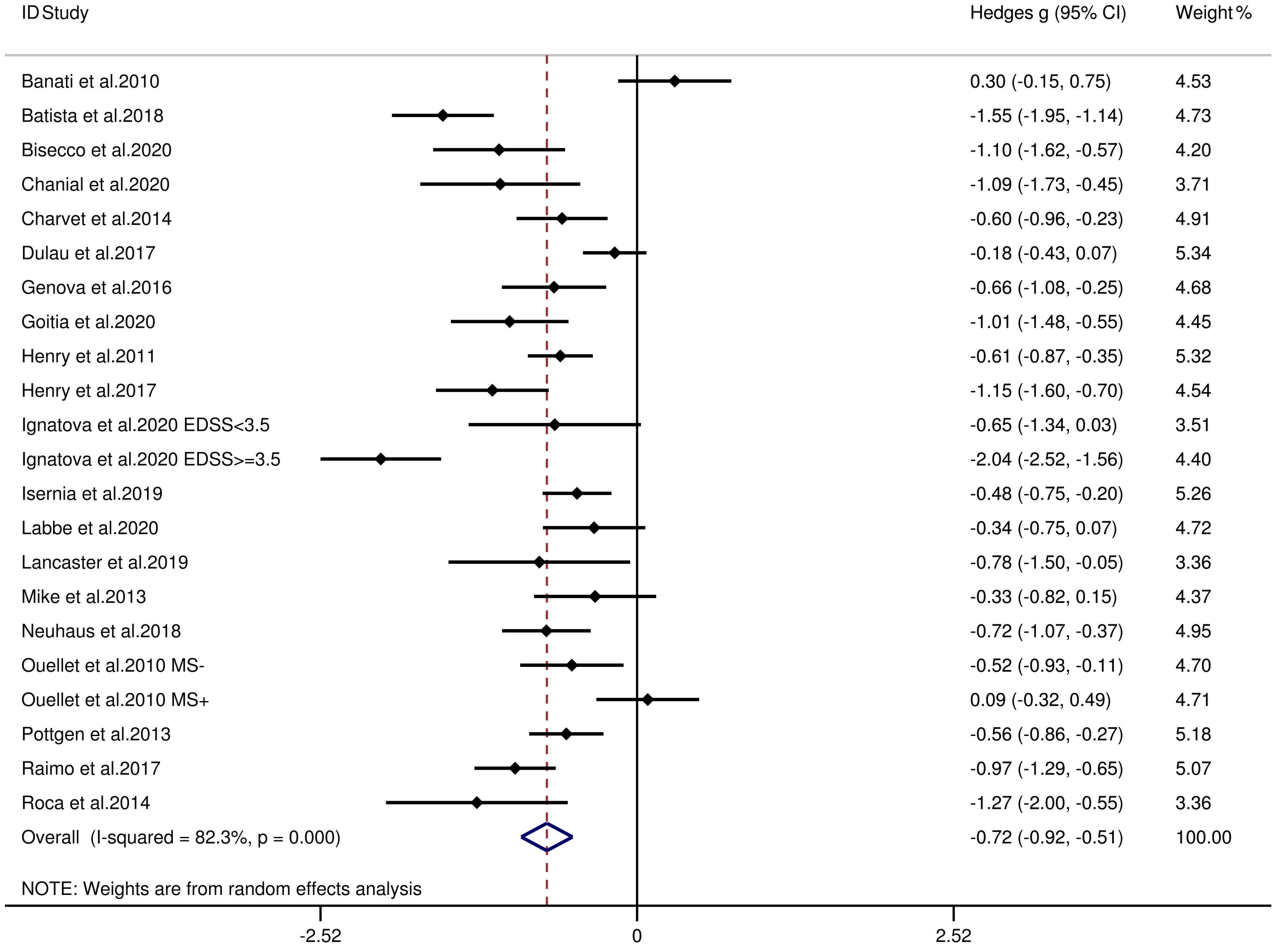
Forest plots showing effect size estimates (Hedges g) for cognitive ToM differences between MS and healthy controls. CI, confidence interval; MS, multiple sclerosis; ToM, theory of mind.

**Figure 5:**
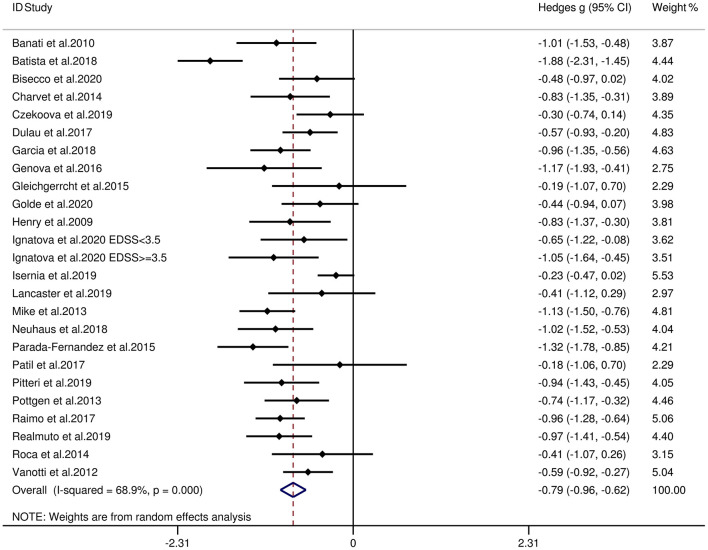
Forest plots showing effect size estimates (Hedges g) for cognitive empathy/affective ToM differences between MS and healthy controls. CI, confidence interval; MS, multiple sclerosis; ToM, theory of mind.

**Figure 6:**
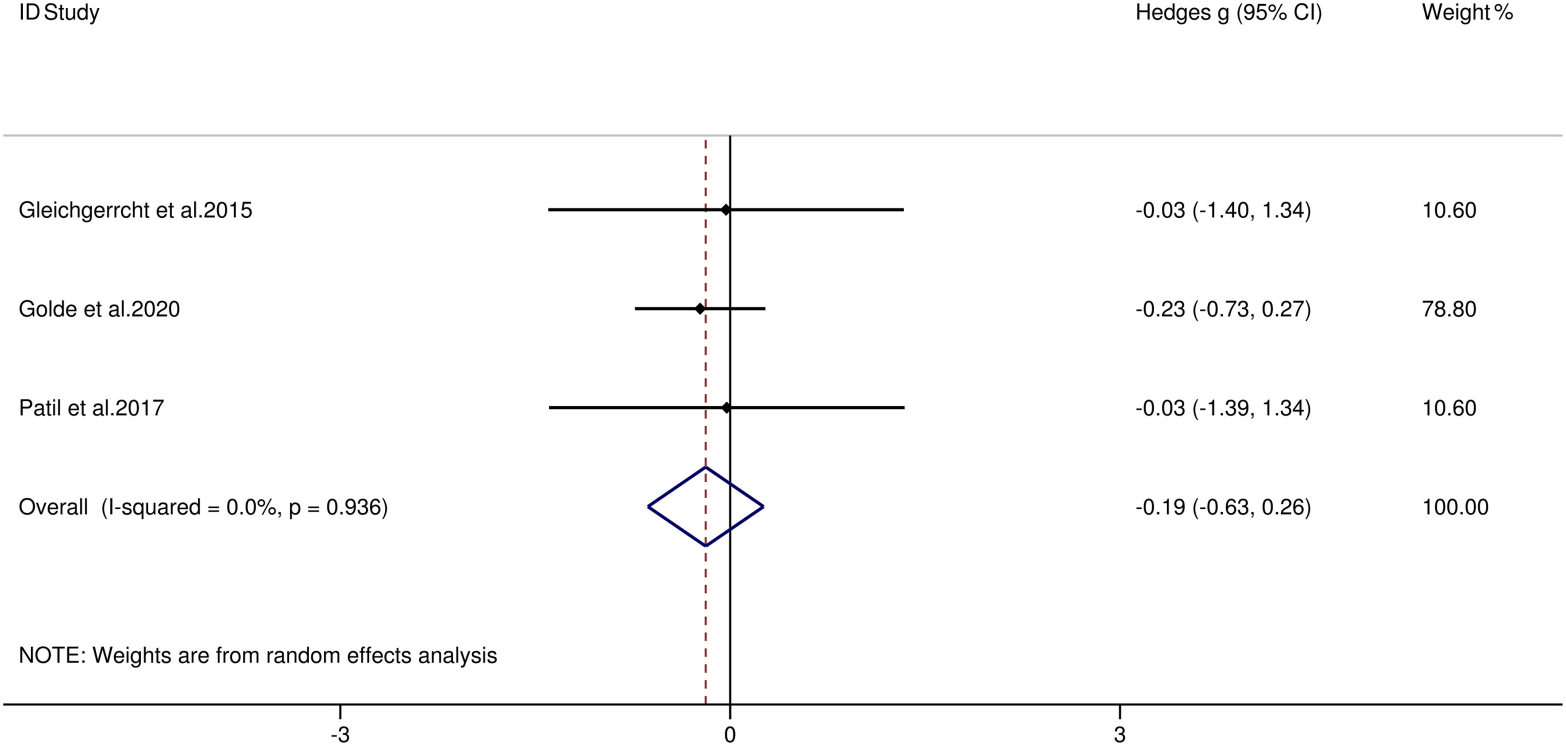
Forest plots showing effect size estimates (Hedges g) for affective empathy differences between MS and healthy controls. CI, confidence interval; MS, multiple sclerosis; ToM, theory of mind.

Table 3 reports the key results from this meta-analysis. Compared to HCs, patients with MS were impaired in overall empathy, with this deficit being moderate in magnitude (*g* = −0.67, 95% CI [−0.84, −0.50], *K* = 27; see Figure 2). Patients with MS were also moderately impaired in their overall ToM ability (*g* = −0.74, 95% CI [−0.88, −0.61], *K* = 34; see Figure 3). Examining the overlapping and distinct subcomponents of these constructs revealed that MS was associated with moderate deficits in cognitive ToM (*g* = −0.72, 95% CI [−0.92, −0.51], *K* = 22; see Figure 4) and cognitive empathy/affective ToM (*g* = −0.79, 95% CI [−0.96, −0.62], *K* = 25; see Figure 5). However, no group differences were evident for affective empathy (*g* = −0.19, 95% CI [−0.63, 0.26], *K* = 3; see Figure 6). There was no heterogeneity across studies for affective empathy (*I*^2^ = 0) and moderate heterogeneity across studies for overall ToM (*I*^2^ = 70.8%), overall empathy (*I*^2^ = 74.1%), and affective ToM/cognitive empathy (*I*^2^ = 68.9%), but there was significant heterogeneity in studies for cognitive ToM (*I*^2^ = 82.3%). Egger's test was not significant for overall empathy, overall ToM, cognitive ToM, or cognitive empathy/affective ToM. Egger's test was only significant for affective empathy (*p* = 0.005). However, a trim-and-fill analysis did not result in imputation of any studies, and the effect size remained similar (*g* = −0.23, 95% CI [–0.63, 0.18]).

**Table 3:** Mean effects for ToM and empathy subcomponents comparing participants with multiple sclerosis against healthy controls and tests for publication bias.

**Subcomponent**	**K**	**N in MS groups**	**N in HCs groups**	**g**	**95% CI**		**Test for Heterogeneity**	**Assess risk of publication bias**	
					**Lower**	**Upper**	***I*^**2**^ statistic,%**	**Egger's test *P*-Value**	**Trim and fill imputed g**
overall empathy	27	1,275	1,110	−0.67	−0.84	−0.50	74.1	0.099	No change
overall ToM	34	1,295	1,208	−0.74	−0.88	−0.61	70.8	0.303	No change
CogToM	22	805	710	−0.72	−0.92	−0.51	82.3	0.062	No change
CogEmp/AffTom	25	972	957	−0.79	−0.96	−0.62	68.9	0.244	No change
AffEmp	3	106	106	−0.19	−0.63	0.26	0	0.005	Similar

*MS, multiple sclerosis; HCs, healthy controls; ToM, theory of mind; CI, confidence interval; CogToM, cognitive ToM; CogEmp/AffToM, cognitive empathy/affective ToM; AffEmp, affective empathy; g, Hedges g; K, the number of studies; N, the number. Trim and fill: look for missing studies to left of mean, using random effects model. Imputed mean is random effects*.

### Empathy and ToM in Patients With RRMS vs. HCs

Table 4 reports the key results from this meta-analysis. Relative to HCs, patients with RRMS exhibited low impairment in overall empathy (*g* = −0.43, 95% CI [−0.57, −0.29], *K* = 17), and moderate impairment in overall ToM ability (*g* = −0.67, 95% CI [−0.82, −0.52], *K* = 20). Examining the overlapping and distinct subcomponents of these constructs revealed that RRMS was associated with significant and large-sized deficits in cognitive ToM (*g* = −0.83, 95% CI [−1.17, −0.50], *K* = 11) and cognitive empathy/affective ToM (*g* = −0.59, 95% CI [−0.77, −0.41], *K* = 15). However, no group differences were evident for affective empathy (*g* = −0.19, 95% CI [−0.63, 0.26], *K* = 3). There was no heterogeneity across studies for affective empathy (*I*^2^ = 0), low heterogeneity across studies for overall empathy (*I*^2^ = 39.1%), and moderate heterogeneity across studies for overall ToM (*I*^2^ = 53.3%) and affective ToM/cognitive empathy (*I*^2^ = 52.2%), but there was significant variation among studies for cognitive ToM (*I*^2^ = 85.4%). Egger's test was not significant for overall ToM, overall empathy, cognitive ToM, or affective ToM/cognitive empathy. Egger's test was only significant for affective empathy. However, a trim-and-fill analysis did not result in imputation of any studies, and the effect size remained similar.

**Table 4:** Mean effects for ToM and empathy subcomponents comparing participants with relapsing-remitting multiple sclerosis against healthy controls and tests for publication bias.

**Subcomponent**	**K**	**N in RRMS groups**	**N in HCs groups**	**g**	**95% CI**		**Test for Heterogeneity**	**Assess risk of publication bias**	
					**Lower**	**Upper**	***I*^**2**^ statistic,%**	**Egger's test *P*-Value**	**Trim and fill imputed g**
Overall empathy	17	858	772	−0.43	−0.57	−0.25	39.1	0.094	No change
Overall ToM	20	758	794	−0.67	−0.82	−0.52	53.3	0.282	No change
CogToM	11	357	466	−0.83	−1.17	−0.50	85.4	0.053	No change
CogEmp/AffTom	15	555	619	−0.59	−0.77	−0.41	52.2	0.172	No change
AffEmp	3	106	106	−0.19	−0.63	0.26	0	0.005	Similar

*RRMS, relapsing-remitting multiple sclerosis; HCs, healthy controls; ToM, theory of mind; CI, confidence interval; CogToM, cognitive ToM; CogEmp/AffToM, cognitive empathy/affective ToM; AffEmp, affective empathy; g, Hedges g; K, the number of studies; N, the number. Trim and fill: look for missing studies to left of mean, using random effects model. Imputed mean is random effects*.

### Empathy and ToM in Patients With Progressive MS vs. HCs

Table 5 reports the key results from this meta-analysis. Relative to HCs, patients with progressive MS exhibited a moderate-sized deficit in overall empathy (*g* = −0.50, 95% CI [−0.73, −0.27], *K* = 4), overall ToM ability (*g* = −0.75, 95% CI [−1.08, −0.41], *K* = 7, cognitive ToM (*g* = −0.72, 95% CI [−1.15, −0.29], *K* = 6), and affective ToM/cognitive empathy (*g* = −0.50, 95% CI [−0.73, −0.27], *K* = 4). No analysis for affective empathy was conducted, as no such studies were included in the meta-analysis. There was no heterogeneity across studies for overall empathy (*I*^2^ = 0) and affective ToM/cognitive empathy (*I*^2^ = 0) and moderate heterogeneity across studies for overall ToM (*I*^2^ = 65%), but there was significant variation among studies for cognitive ToM (*I*^2^ = 75.5%). Egger's test was not significant for overall ToM, overall empathy, cognitive ToM, or affective ToM/cognitive empathy.

**Table 5:** Mean effects for ToM and empathy subcomponents comparing participants with progressive multiple sclerosis against healthy controls and tests for publication bias.

**Subcomponent**	**K**	**N in progressive MS groups**	**N in HCs groups**	**g**	**95% CI**		**Test for Heterogeneity**	**Assess risk of publication bias**	
					**Lower**	**Upper**	***I*^**2**^ statistic,%**	**Egger's test *P*-Value**	**Trim and fill imputed g**
overall empathy	7	61	101	−0.50	−0.73	−0.27	0	0.366	No change
overall ToM	4	124	149	−0.75	−1.08	−0.41	64.9	0.091	No change
CogToM	6	107	134	−0.72	−1.15	−0.29	75.5	0.196	No change
CogEmp/AffTom	4	61	101	−0.50	−0.73	−0.27	0	0.366	No change

*Progressive MS, progressive multiple sclerosis; HCs, healthy controls; ToM, theory of mind; CI, confidence interval; CogToM, cognitive ToM; CogEmp/AffToM, cognitive empathy/affective ToM; AffEmp, affective empathy; g, Hedges g; K, the number of studies; N, the number. Trim and fill: look for missing studies to left of mean, using random effects model. Imputed mean is random effects*.

### Empathy and ToM in Patients With RRMS vs. Patients With Progressive MS

Table 6 reports the key results from this meta-analysis. Relative to patients with progressive MS, patients with RRMS showed no difference in overall empathy (*g* = 0.21, 95% CI [−0.23, 0.65], *K* = 2), overall ToM (*g* = 0.11, 95% CI [−0.09, 0.31], *K* = 3), cognitive ToM (*g* = 0.05, 95% CI [−0.14, 0.24], *K* = 3), and affective ToM/cognitive empathy (*g* = 0.21, 95% CI [−0.23, 0.65], *K* = 2). No analysis for affective empathy was conducted, as no such studies were included in this meta-analysis. There was low heterogeneity across studies for overall ToM (*I*^2^ = 23.7%) and cognitive ToM (*I*^2^ = 15.4%) and moderate heterogeneity across studies for overall empathy (*I*^2^ = 63.2%) and cognitive empathy/affective ToM (*I*^2^ = 63.2%). Egger's test was not significant for overall ToM and cognitive ToM.

**Table 6:** Mean effects for ToM and empathy subcomponents comparing participants with relapsing-remitting multiple sclerosis against progressive multiple sclerosis and tests for publication bias.

**Subcomponent**	**K**	**N in RRMS groups**	**N in progressive MS groups**	**g**	**95% CI**		**Test for Heterogeneity**	**Assess risk of publication bias**	
					**Lower**	**Upper**	***I*^**2**^ statistic,%**	**Egger's test *P*-Value**	**Trim and fill imputed g**
Overall empathy	2	56	46	0.21	−0.23	0.65	63.2		
Overall ToM	3	87	77	0.11	−0.09	0.31	23.7	0.303	No change
CogToM	3	87	77	0.05	−0.14	0.24	15.4	0.062	No change
CogEmp/AffTom	2	56	46	0.21	−0.23	0.65	63.2		

*RRMS, relapsing-remitting multiple sclerosis, progressive MS progressive multiple sclerosis; ToM, theory of mind; CI, confidence interval; CogToM, cognitive ToM; CogEmp/AffToM, cognitive empathy/affective ToM; AffEmp, affective empathy; g, Hedges g; K, the number of studies; N, the number. Trim and fill: look for missing studies to left of mean, using random effects model. Imputed mean is random effects*.

### Meta-Regression Analyses

Meta-regression analyses showed that the included variables did not account for significant variance across studies. The variables (age, sex, education level, disease duration, EDSS score, quality assessment score, severity of depression, and severity of anxiety) did not account for significant variance in overall empathy(*p* = 0.871, 0.218, 0.582, 0.996, 0.712, 0.318, 0.671, and 0.871, respectively), overall ToM (*p* = 0.825, 0.341, 0.832, 0.245, 0.527, 0.535, 0.068, and 0.224, respectively), cognitive ToM (*p* = 0.961, 0.418, 0.89, 0.997, 0.831, 0.098, 0.423, NA, respectively), or cognitive empathy/affective ToM (*p* = 0.548, 0.516, 0.61, 0.634, 0.549, 0.589, 0.48, and 0.872, respectively). No meta-regression analyses were conducted for the severity of anxiety in cognitive ToM, as fewer than 10 studies contributed to the data for this subcomponent.

## Discussion

To our knowledge, this was the first meta-analysis to investigate the patterns of empathy and ToM functioning in patients with MS. The meta-analysis included 33 studies, with combined samples of 1,568 individuals with MS and 1,283 HCs. Relative to the HC group, the MS group showed moderate impairments in both overall empathy (*g* = −0.67) and overall ToM (*g* = −0.74). Among the overlapping and distinct subcomponents of these constructs, MS was associated with moderate impairment in cognitive ToM (*g* = −0.72) and cognitive empathy/affective ToM (*g* = −0.79), but no significant difference was found in affective empathy. Subgroup analyses showed that compared with the HCs, patients with RRMS and progressive MS were both impaired in overall empathy, overall ToM, cognitive ToM, and cognitive empathy/affective ToM, and there was no statistical difference between RRMS and progressive MS in the degree of impairment. Meta-regression analysis indicated that the examined variables (age, sex, education level, disease duration, EDSS score, quality assessment score, severity of depression, and severity of anxiety) did not affect the magnitude of the effect sizes observed.

For overall empathy, a moderate effect size was found (*g* = −0.67). When focusing on the subcomponents of empathy, patients with MS were found to have moderate impairment in cognitive empathy; however, there was no difference in affective empathy. The quantitative findings support the conclusions of previous qualitative studies, indicating that cognitive and affective empathy are separate and have different requirements for effortful processing (81, 83, 90). Specifically, cognitive empathy/affective ToM, requiring attention and time, is a slow and laborious process, while affective empathy, operating with minimal conscious awareness, is an automatic and spontaneous response (95). Therefore, these two empathic components may present different challenges for patients with MS. As affective empathy has low cognitive requirements, it might be expected that this ability remains relatively preserved in MS. This disconnection between the cognitive and affective subcomponents of empathy has been confirmed in several other neurological diseases. For example, in patients with Alzheimer's disease, there was a moderate-sized deficit in cognitive empathy/affective ToM, but no impairment in affective empathy (96). Patients with Parkinson's disease, compared to HCs, PD had significant impairment in cognitive empathy/affective ToM, but no group differences were identified in affective empathy (97). However, the findings should be interpreted with caution in this meta-analysis due to the limited number of included studies contributing to the effect size of affective empathy (*K* = 3).

The results pertaining to overall ToM impairment supported the findings of Bora et al. (32) and Cotter et al. (33), showing that overall ToM is moderately impaired in MS. When considering the sub-components of ToM, some previous studies have suggested that the domains of cognitive and affective ToM are dissociated, and the function of cognitive empathy/affective ToM in MS is conserved, but cognitive ToM is impaired (74, 85, 88, 93). However, findings from the current quantitative meta-analysis do not support this suggestion, which showed that patients of MS had moderate impairment in both cognitive and affective ToM and their degrees of defect were close (*g* = −0.72 and *g* = −0.79, respectively). This impairment may be related to white matter (WM) damage in MS. On the macro-structure, ToM impairment is associated with T1 and T2 lesions (65, 71, 88); on the microstructure, ToM impairment is shown to be related to the disconnection with the social brain network caused by diffuse normal-appearing white matter damage in MS, especially in tracts of limbic pathways (uncinate fasciculus, fornix) and callosal interhemispheric fibers (corpus callosum, tapetum) (72), which play a key role in social and communication skills or emotional processing (98–100). In addition, gray matter (GM) pathology is considered to have an important role in ToM impairment. GM atrophy was found in the cingulate, orbitofrontal, cerebellar cortex, and insula decreased was found (65, 66, 72), which are involved in cognitive and affective ToM network (101). Several studies based on magnetic resonance imaging (MRI) have found that amygdala atrophy is the main predictor of ToM impairment in MS (66, 72). Besides, one resting-state functional MRI study found that there was an association between ToM impairment and functional connectivity changes in the default mode network, executive network, and limbic network in MS (74).

In the subgroup meta-analyses, the results showed that compared with HCs, patients with RRMS and progressive MS were impaired in overall empathy, overall ToM, cognitive ToM, and cognitive empathy/affective ToM, and there was no statistical difference between RRMS and progressive MS in overall empathy, overall ToM, cognitive ToM, and cognitive empathy/affective ToM. This result is inconsistent with the previous quantitative results of Bora et al., which indicated that social cognition tended to be more impaired in progressive MS in comparison to RRMS. However, it should be noted that the aforementioned Bora et al. study calculated a social cognition score based on numerous very different ToM tasks and facial emotion recognition tasks (another core domain of social cognition). Besides, due to the limited number of included studies contributing to the comparison between RRMS and progressive MS (*K* = 3 in this study, *K* = 5 in the study by Bora et al.), we should cautiously interpret the results.

Our meta-analysis findings may contribute to the development of cognitive rehabilitation for MS. Several studies have shown that cognitive rehabilitation intervention may have a positive impact on MS symptoms (102–104). In particular, depression symptoms, anxiety, fatigue, pain, physical vitality, and sleep quality improved significantly after most of the cognitive rehabilitation intervention (104–107). Besides, studies have shown that cognitive rehabilitation can improve the cognitive function in patients with MS, mainly focusing on general cognitive functions such as memory, executive function, attention, and processing speed (102, 104, 108–112). However, there are few studies about how the cognitive interventions affect social cognitive in MS. Our meta-analytic findings can broaden the theoretical understanding of MS, which may help improve or formulate cognitive intervention strategies.

## Limitations

The current meta-analysis has some limitations. First, although 33 studies were included in this meta-analysis, only three contributed to the mean effect size for affective empathy between patients with MS and HCs. In addition, only three studies provided data comparing RRMS and progressing MS; hence, more research in this area is needed in the future. Second, we only included cross-sectional studies, while more longitudinal studies are needed to investigate the dynamic changes in empathy and ToM function in patients of MS. Third, although we investigated some demographic and clinical variables (including age, sex, education level, disease duration, EDSS scores, severity of depression, and severity of anxiety) that may affect empathy and ToM function, other factors [such as prior substance abuse or some other behavioral symptoms (including apathy, inflexible, obsessive, sometimes with flattened affect, suspiciousness, etc.)] were not examined due to the limited data available in the original studies (67, 113, 114). Further studies are required to comprehensively elucidate the potential effects of these factors on empathy- and ToM-associated features in MS. Fifth, there was heterogeneity between the individual tasks for the assessment of ToM or empathy, and further development of standardized batteries for ToM/empathy assessment in MS is needed. For example, the Measurement and Treatment Research to Improve Cognition in Schizophrenia Cognition in Schizophrenia (MATRICS) Consensus Cognitive Battery (MCCB) (115), which makes it possible to standardize the evaluation of cognitive outcomes in schizophrenia, may also be adapted for MS.

## Conclusions

The results of this meta-analysis suggest that patients with MS exhibited moderate impairment in broad constructs of ToM and empathy and the ToM subcomponents (cognitive ToM and affective ToM/cognitive empathy), but no significant impairment in affective empathy. These quantitative results suggest a differential impairment of the core aspects of social cognitive (including empathy and ToM) processing in patients with MS, which may greatly inform the development of structured social cognitive interventions in MS.

## Data Availability Statement

The original contributions presented in the study are included in the article/supplementary material, further inquiries can be directed to the corresponding author/s.

## Author Contributions

ZY and GW: study design and critical revision of the manuscript. XL, XZ, QL, PZ, JZ, and PP: analysis and interpretation of data. XL and XZ: drafting of the manuscript. All authors: approval of the final version for submission.

## Conflict of Interest

The authors declare that the research was conducted in the absence of any commercial or financial relationships that could be construed as a potential conflict of interest.
